# Exploiting Impact of Hardware Impairments in NOMA: Adaptive Transmission Mode in FD/HD and Application in Internet-of-Things

**DOI:** 10.3390/s19061293

**Published:** 2019-03-14

**Authors:** Chi-Bao Le, Dinh-Thuan Do, Miroslav Voznak

**Affiliations:** 1 Faculty of Electronics Technology, Industrial University of Ho Chi Minh City (IUH), Ho Chi Minh City 700000, Vietnam; lechibao@iuh.edu.vn; 2 Wireless Communications Research Group, Faculty of Electrical and Electronics Engineering, Ton Duc Thang University, Ho Chi Minh City 700000, Vietnam; 3 VSB—Technical University of Ostrava, 17. listopadu 15, 708 00 Ostrava, Czech Republic; miroslav.voznak@vsb.cz

**Keywords:** non-orthogonal multiple access, full-duplex, outage probability

## Abstract

In this paper, a cooperative non-orthogonal multiple access (NOMA) system is studied for the Internet-of-Things (IoT) in which a master node intends to serve multiple client nodes. The adaptive transmission strategy is proposed at the relay node, i.e., the relay can be half-duplex (HD) and/or full duplex (FD). In practical terms, numerous low-cost devices are deployed in such IoT systems and it exhibits degraded performance due to hardware imperfections. In particular, the effects of hardware impairments in the NOMA users are investigated. Specifically, the closed-form expressions are derived for the outage probability. Moreover, the ergodic capacity is also analysed. This study also comparatively analyzes the orthogonal multiple access (OMA) and NOMA with HD and/or FD relaying. The numerical results are corroborated through Monte Carlo simulations.

## 1. Introduction

The non-orthogonal multiple access (NOMA) technique has evolved as one of the potential technologies for efficient utilization of resources in wireless communication systems [1,2,3]. Unlike the orthogonal multiple-access (OMA) scheme, larger number of users can be connected to a wireless network concurrently in a NOMA scenario [4]. Furthermore, the NOMA also improves throughput and coverage in the uplink and downlink of a wireless network by incorporate with relaying schemes reported in [5,6]. For all these advantages, NOMA is explored in the recent literature as the candidate for upcoming wireless communication systems such as 5G and beyond [1,2,3].

Recent works in the open literature have extensively analyzed NOMA systems. For example, Wang et al. in [7] investigated the power allocation problem for sum rate improvement in a NOMA system. A cooperative NOMA scheme for full-duplex (FD) device-to-device communication has been studied in [8], in which the improvement in term of the outage performance for NOMA users was evaluated. To improve the spectrum efficiency, the novel pattern regarding division multiple access was suggested in [9]. In [10], the optimum power distribution was proposed to maximize the energy effectiveness of NOMA systems. To realize spectrum sharing in cognitive radio networks, NOMA can be deployed as a promising approach [11]. Especially, the authors in [12] have shown a novel cognitive radio in which a NOMA-assisted secondary transmitter was considered. A two-stage relay selection is introduced in [13] and the authors investigated the outage performance of NOMA system equipped with a decode-and-forward (DF) relaying technique. In [14], an optimum joint user and relay selection procedure was suggested to perform the amplify-and-forward (AF) relaying in a cooperative NOMA network. More recently, while considering the location of relay in NOMA systems, some stochastic geometry models were applied to evaluate the performance. In [15], to improve the security of a random network, large-scale NOMA systems is examined in term of the physical layer security. In addition, the authors in [15] also proposed a secure zone around the source node.

The considered emerging techniques including NOMA and FD have been explored for next generation wireless networks. By allowing the radio signal to simultaneously transmit and receive on the same frequency channel, the spectral efficiency in FD communication can be enhanced two times [16,17,18,19]. It is worth noting that signal leakage is considered as the main challenge for realizing the FD communication which leads to the self-interference (SI) and considerably destroys the performance. In other trend of research, full-duplex non-orthogonal multiple access (FD-NOMA) is studied with co-channel interference (CCI) [20]. The ergodic sum capacity is examined in the time sharing (TS) cooperative NOMA with HD/FD scheme [21]. FD can also be used to realize simultaneous NOMA uplink and downlink transmissions, such as in [22], both power allocation and subcarrier allocation have been investigated in such FD-NOMA scenario, and its multi-cell extension has been considered in [23].

### 1.1. Related Works

Furthermore, the influence of hardware impairment noise on different categories of wireless networks has been well explored [24,25,26,27,28,29]. In general, hardware impairments have a harmful influence on the attainable performance as in relaying networks [25,26,27,28,29]. As a result, certain calibration techniques at the transmitter or/and compensation algorithms at the receivers are required. The system performance degradation caused by hardware impairments can be practically alleviated by using the scheme proposed in [24]; however, the residual hardware impairments (RHI) affect the performance of systems [24].

The effects of hardware impairment such as in-phase/quadrature-phase imbalance (IQI) on the performance are studied in NOMA-based single carrier (SC) and multi-carrier (MC) systems under different underlying systems’ parameters [30]. Serveral important results were obtained in [30] including, the impairment situation, the required rates, the power allocation factors and the order of the NOMA users and then the level of performance degradation caused by IQI is evaluated. The authors in [31] presented the exact expression for outage probability in closed-form. Main metrics including outage probability and ergodic capacity are examined in recent work [32]. While only ergodic capacity is evaluated in NOMA model in [33]. In addition, the achievable outage performance of both users and ergodic sum capacity are studied in full-duplex FD- NOMA system with dual users [34].

### 1.2. Contributions and Organization

As discussed above, residual transceiver hardware impairments are the main reasons which decline the performance of the conventional NOMA-based relaying networks [30,31]. For cooperative relaying NOMA networks, the HD one is proposed for downlink in [32] and for uplink in [33]. To the best knowledge of the authors, NOMA-based full-duplex relaying scenario with hardware impairments is not considered in the aforementioned works. This motives us to derive the expressions for outage probability and ergodic capacity. Precisely, cumulative residual hardware impairments are examined to evaluate the performance of FD NOMA relaying network. This is an interesting system model for future applications as it can achieve higher bandwidth compared with HD NOMA. We highlight the influence of hardware impairment (HI) for such FD NOMA relaying network. The analysis exploits the residual impairment scenario to provide significant limitation of practical NOMA deployments. Such deployment can be provided to design of Internet of Things where massive connections need be served simultaneously. The main contributions through this work are as follows:

- Considering the cooperative relaying NOMA system as in [34], the outage performance and ergodic capacity for the near user and the far users under impacts of residual hardware impairment (RHI) are examined under Rayleigh fading environment.
- We also propose two adaptive schemes considering the trade-offs between FD and HD NOMA relaying and between NOMA and OMA FD relaying, respectively.
- Comparison study is performed for two typical users, i.e., near user and far user in NOMA, for both outage performance and ergodic capacity. These performance metric are employed by controlling level of hardware noise, residual interference due to FD mode to adapt requirements of wireless system.
- Extensive Monte Carlo simulation results are presented in order to corroborate the derived exact and asymptotic expressions.

The remaining section of this study is organized as follows. In Section 2, the system model for NOMA-based DF relaying networks with RHI, are presented. We derive the exact expressions for outage probability and integral form for Ergodic capacity for FD and HD cooperative NOMA in Section 3 and Section 4, respectively. In Section 5, the mode selection scheme is propose and the analytical expressions of the outage probability for each user are obtained. Section 6 presents simulation results to verify our theoretical analysis. Finally, Section 7 concludes the paper.

**Notation:**$F_{X}\left(x\right)$ and $f_{X}\left(x\right)$ represent the cumulative distribution function (CDF) and probability density function (PDF) of random variable (R.V) *X*, respectively. $x∼CN\left(0,b\right)$ means that *x* is a complex normal distributed random variable with zero mean and variance *b*.

## 2. System Model

**Figure 1:**
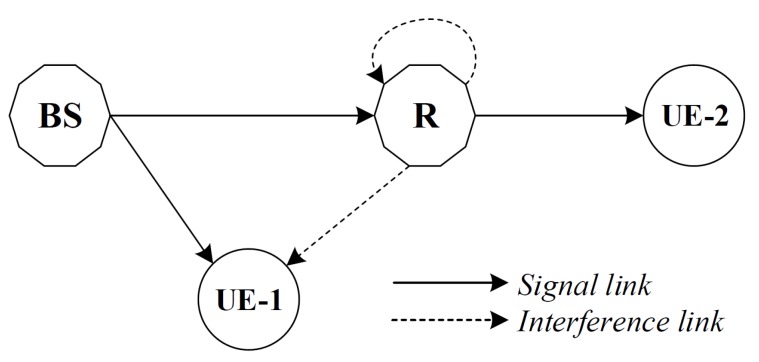
The block diagram of considered system model.

**Figure 2:**
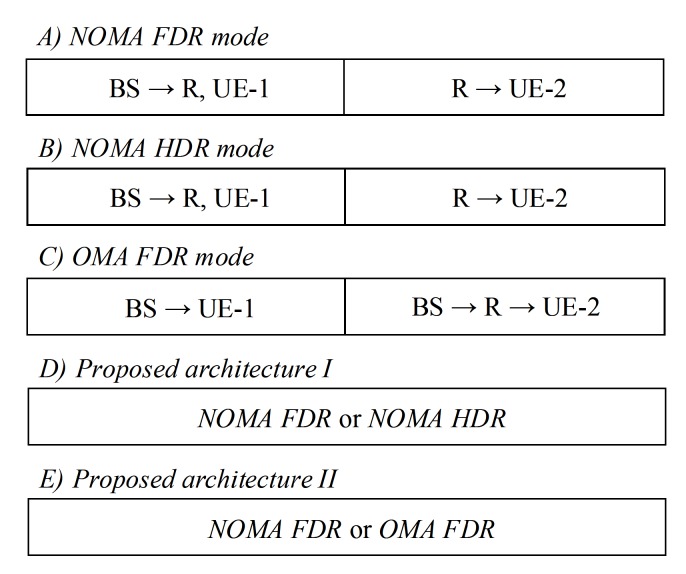
Time slots are allocated for several transmission modes.

In this paper, as in Figure 1 we consider the case of down link NOMA in Internet of Things (IoT) system where two client users including near user denoted as UE–1 and far user denoted as UE–2. Such model is very popular in applications deployment of IoT where massive connections are served simultaneously. Both the users are served by a master node operated as base station (BS) at the same time and frequency, but with different power levels. Due to the far distance between UE–2 and BS, we assume FD cooperative relaying, where FD relay R assists the communication between BS and UE–2. As an illustration in Figure 2, regarding time slots are assigned for these traditional models as NOMA FDR mode, NOMA HDR mode, OMA FDR mode, they require to allocate two time slots for their transmission while our proposed architecture need only time slot for hybrid scheme. These transmission modes are described in many works [32,34]. It is noted that power domain multiplexing in NOMA can be efficiently performed by deploying superposition coding at the receivers.

### 2.1. Transceiver Hardware Impairment Model

Considering that the transmitter *A* conveys a unit variance symbol *x* to the receiver *B* with transmit power $P_{x}$ via channel *h*, the baseband signal received at *B*, i.e., $y_{B}$, can be succinctly expressed as [26,35],

(1) $$\begin{array}{c} y_{B}=h\left(x+\tau_{x}\right)+\nu_{y}+w_{B}, \end{array}$$

where $\tau_{x}∼CN\left(0,\kappa_{x}^{2}P_{x}\right)$, $\nu_{y}∼CN\left(0,\vartheta_{y}^{2}P_{x}{\left|h\right|}^{2}\right)$ represent the noise terms of imperfect transmitter and receiver hardware, respectively. Besides, $\kappa_{x},\vartheta_{y}\in \left[0,0.175\right]$ are imperfect levels of corresponding hardware noise. While $w_{B}$ is additive noise at *B*, and ${\left|h\right|}^{2}$ represents the channel power gain.

### 2.2. Signal Model for NOMA FD Relaying Networks

We assume a dual user NOMA scheme in which the BS directly transmits the message to the UE–1 while UE–2 is assisted by a FD relay with a DF relaying protocol as we showed in Figure 1.

#### 2.2.1. UE–1 Analysis

In the *n*–th time slot, the BS sends a superimposed message to both UE–1 and UE–2 given by

(2) $$s\left[n\right]=\sqrt{a_{1}P_{\mathrm{bs}}}x_{u1}\left[n\right]+\sqrt{a_{2}P_{\mathrm{bs}}}x_{u2}\left[n\right],$$

where $a_{1}$ and $a_{2}$ denote the fraction of allocated powers for UE–1 and UE–2 signals, respectively, with $a_{1}+a_{2}=1$. Moreover, $x_{u1}∼CN\left(0,1\right)$, $x_{u2}∼CN\left(0,1\right)$ and $s∼CN\left(0,P_{\mathrm{bs}}\right)$ are the messages from UE–1, UE–2 and NOMA data symbol, respectively.

According to NOMA cooperation principle, the message of UE–2 can be decoded by UE–1 whether it is successfully decoded or not and we consider $x_{2}\left[n-\hat{n}\right]$ as message at UE–1.

The received signal at UE–1 is given by exploiting known interference cancellation and can be written as [34],

(3) $$\begin{array}{cc} y_{u1}\left[n\right]= & h_{\mathrm{bs},u1}\left(s\left[n\right]+\tau_{\mathrm{bs}}\right)+\sqrt{P_{\mathrm{re}}}f_{\mathrm{re},u1}\left(x_{2}\left[n-\hat{n}\right]+\tau_{\mathrm{re}}\right)+\nu_{u1}+w_{u1}\left[n\right] \end{array}$$

where $\tau_{\mathrm{bs}}∼CN\left(0,\kappa_{\mathrm{bs}}^{2}P_{\mathrm{bs}}\right)$, $\tau_{\mathrm{re}}∼CN\left(0,\kappa_{\mathrm{re}}^{2}P_{\mathrm{re}}\right)$ and $\nu_{u1}∼CN\left(0,\vartheta_{u1}^{2}\left(P_{\mathrm{bs}}{\left|h_{\mathrm{bs},u1}\right|}^{2}+P_{\mathrm{re}}{\left|f_{\mathrm{re},u1}\right|}^{2}\right)\right),$ are RHI noise of BS transmitter, relay transmitter and UE–1 receiver, respectively. In addition, $h_{\mathrm{bs},u1}∼\mathrm{CN}\left(0,\lambda_{\mathrm{bs},u1}\right)$ is the channel fading between the BS and UE–1, $f_{\mathrm{re},u1}∼\mathrm{CN}\left(0,\xi_{\mathrm{re},u1}\lambda_{\mathrm{re},u1}\right)$ is the CCI channel coefficient from relay to UE–1, and the parameter $\xi_{\mathrm{re},u1}\in \left[0,1\right]$ represents the residual interference grade. Specifically, $\xi_{\mathrm{re},u1}=0$ indicates impeccable interference cancellation. Further, $P_{\mathrm{re}}$ is relay transmission power and $w_{u1}$ is the additive white Gaussian noise (AWGN) at UE–1 with zero mean and variance $N_{0}$. We define the signal-to-noise ratio (SNR) at BS and relay as $SNR_{\mathrm{bs}}=P_{\mathrm{bs}}/N_{0}$ and $SNR_{\mathrm{re}}=P_{\mathrm{re}}/N_{0}$, respectively. Further, we denote $\varepsilon_{1}=a_{1}SNR_{\mathrm{bs}}$, $\varepsilon_{2}=SNR_{\mathrm{bs}}\left(\kappa_{\mathrm{bs}}^{2}+\vartheta_{u1}^{2}\right)$, $\varepsilon_{3}=SNR_{\mathrm{re}}\left(1+\kappa_{\mathrm{re}}^{2}+\vartheta_{u1}^{2}\right)$, $\varepsilon_{4}=a_{2}SNR_{\mathrm{bs}}$. If UE–1 can decode the message of UE–2 completely, i.e., the achievable rate of $R_{2}$ satisfies this condition $C_{\mathrm{bs}\to u1}^{u2}=\log_{2}\left(1+\gamma_{\mathrm{bs}\to u1}^{u2}\right)>R_{2}$ where $\gamma_{\mathrm{bs}\to u1}^{u2}$ given by

(4) $$\gamma_{\mathrm{bs}\to u1}^{u2}=\frac{\varepsilon_{4}{\left|h_{\mathrm{bs},u1}\right|}^{2}}{\left(\varepsilon_{1}+\varepsilon_{2}\right){\left|h_{\mathrm{bs},u1}\right|}^{2}+\varepsilon_{3}{\left|{\hat{f}}_{\mathrm{re},u1}\right|}^{2}+1},$$

is effective SINR of UE–2 detected at UE–1, then the achievable rate at UE–1 can hence be obtained as

(5) $$C_{\mathrm{bs}\to u1}^{u1}=\log_{2}\left(1+\gamma_{\mathrm{bs}\to u1}^{u1}\right),$$

with SINR of UE–1 using for successive interference cancellation (SIC) in (5) can be expressed as

(6) $$\gamma_{\mathrm{bs}\to u1}^{u1}=\frac{\varepsilon_{1}{\left|h_{\mathrm{bs},u1}\right|}^{2}}{\varepsilon_{2}{\left|h_{\mathrm{bs},u1}\right|}^{2}+\varepsilon_{3}{\left|f_{\mathrm{re},u1}\right|}^{2}+1}.$$

#### 2.2.2. UE–2 Analysis

The received signal at the relay can be expressed as

(7) $$\begin{array}{cc} y_{\mathrm{re}}\left[n\right]= & h_{\mathrm{bs},\mathrm{re}}\left(s\left[n\right]+\tau_{\mathrm{bs}}\right)+f_{\mathrm{re},\mathrm{re}}\left(\sqrt{P_{\mathrm{re}}}x_{2}\left[n-\tau \right]+\tau_{\mathrm{re}}\right)+\nu_{\mathrm{re}}+w_{\mathrm{re}}\left[n\right], \end{array}$$

where $\nu_{\mathrm{re}}∼CN\left(0,\vartheta_{\mathrm{re}}^{2}\left[P_{\mathrm{bs}}{\left|h_{\mathrm{bs},\mathrm{re}}\right|}^{2}+P_{\mathrm{re}}{\left|f_{\mathrm{re},\mathrm{re}}\right|}^{2}\right]\right)$ denotes receiver RHI noise, $\tau_{\mathrm{bs}},\tau_{\mathrm{re}}$ are defined below (3) and $h_{\mathrm{bs},\mathrm{re}}∼CN\left(0,\lambda_{\mathrm{bs},\mathrm{re}}\right)$ is the channel coefficient from the BS to relay. Further, $f_{\mathrm{re},\mathrm{re}}∼CN\left(0,\xi_{\mathrm{re},\mathrm{re}}\lambda_{\mathrm{re},\mathrm{re}}\right)$ is the channel coefficient for the relay SI link with $\xi_{\mathrm{re},\mathrm{re}}\in \left[0,1\right]$ represents the residual SI grade. $w_{\mathrm{re}}$ is the AWGN at the relay with zero mean and variance $N_{0}$.

The relay attempts to decode the message forwarded to UE–2 while treating the signal of UE–1 as interference. The SINR used to decode UE–2 message at the relay is thus, determined as (8)

(8) $$\gamma_{\mathrm{bs}\to \mathrm{re}}^{u2}=\frac{\phi_{1}{\left|h_{\mathrm{bs},\mathrm{re}}\right|}^{2}}{\phi_{2}{\left|h_{\mathrm{bs},\mathrm{re}}\right|}^{2}+\phi_{3}{\left|{\hat{f}}_{\mathrm{re},\mathrm{re}}\right|}^{2}+1},$$

where $\phi_{1}=a_{2}SNR_{bs}$, $\phi_{2}=\left(a_{1}+\kappa_{bs}^{2}+\vartheta_{re}^{2}\right)SNR_{bs}$, and $\phi_{3}=SNR_{re}\left(1+\kappa_{re}^{2}+\vartheta_{re}^{2}\right)$

Hence, the achievable rate at UE–2 can be expressed as

(9) $$C_{\mathrm{bs}\to \mathrm{re}}^{u2}=\log_{2}\left(1+\gamma_{\mathrm{bs}\to \mathrm{re}}^{u2}\right).$$

Finally, the received signal at UE–2 is given by

(10) $$\begin{array}{cc} y_{u2}\left[n\right]= & \sqrt{P_{re}}h_{\mathrm{re},u2}\left(x_{2}\left[n-\hat{n}\right]+\tau_{\mathrm{re}}\right)+\nu_{u2}+w_{u2}\left[n\right], \end{array}$$

where $\nu_{u2}∼CN\left(0,\vartheta_{u2}^{2}P_{\mathrm{re}}{\left|h_{\mathrm{re},u2}\right|}^{2}\right)$ is the receiver HI noise at UE–2. Moreover, $h_{\mathrm{re},u2}∼CN\left(0,\lambda_{\mathrm{re},u2}\right)$ is the channel coefficient between the relay and UE–2, and $n_{u2}∼CN\left(0,N_{0}\right)$ is the AWGN at the UE–2.

Hence, the achievable rate for the relay to UE–2 channel is given by

(11) $$C_{\mathrm{re}\to u2}^{u2}=\log_{2}\left(1+\gamma_{\mathrm{re}\to u2}^{u2}\right)$$

where

$$\gamma_{\mathrm{re}\to u2}^{u2}=\frac{\phi_{4}{\left|h_{\mathrm{re},u2}\right|}^{2}}{\phi_{5}{\left|h_{\mathrm{re},u2}\right|}^{2}+1}$$

is the SINR at UE–2 with $\phi_{4}=SNR_{re}$, $\phi_{5}=\left(\kappa_{re}^{2}+\vartheta_{u2}^{2}\right)SNR_{re}$.

In addition, since $x_{2}\left[n-\hat{n}\right]$ need to be decoded at UE–1 for SIC, the achievable rate for UE–2 can be evaluated as

(12) $$C_{u2}=\log_{2}\left(1+\min\left\{\gamma_{\mathrm{bs}\to \mathrm{re}}^{u2},\gamma_{\mathrm{re}\to u2}^{u2}\right\}\right).$$

## 3. NOMA with Full-Duplex Cooperative Relaying System

### 3.1. Outage Probability Analysis

In this section, the outage performance analysis of the FD cooperative NOMA scheme under hardware impairment condition is presented.

**Lemma** **1.** *Firstly, we define a lemma which is useful for further analysis. Since X, Y have a exponential distribution with zero mean and variance $\lambda_{X}$ and $\lambda_{Y}$, respectively. Then, the CDF of $Z=aX/\left(bX+cY+1\right)$ with a,b,c>0 is given by,*

(13) $$F_{Z}\left(t\right)=\left\{\begin{array}{cc} 1, & t\geq a/b\\ 1-\exp\left(\frac{-t}{\left(a-bt\right)\lambda_{X}}\right)\times {\left(1+\frac{ct\lambda_{Y}}{\left(a-bt\right)\lambda_{X}}\right)}^{-1}, & t<a/b \end{array}\right.$$

**Proof.**  See Appendix A. □

#### 3.1.1. Outage Probability of UE-1 in FD Mode

As discussed earlier, the UE–1 experiences outage when either the UE–1 could not detect its own message or the message received from UE–2. The outage probability of UE–1 thus, can be expressed as

(14) $$\begin{array}{cc} {\mathrm{OP}}_{u1}^{FD}= & \mathrm{Pr}\left(C_{\mathrm{bs}\to u1}^{u2}<R_{2}\cup C_{\mathrm{bs}\to u1}^{u1}<R_{1}\right)\\ = & 1-\mathrm{Pr}\left(\log_{2}\left(1+\gamma_{\mathrm{bs}\to u1}^{u2}\right)\geq R_{2}\right.\cap \left.\log_{2}\left(1+\gamma_{\mathrm{bs}\to u1}^{u1}\right)\geq R_{1}\right). \end{array}$$

**Proposition** **1.** *Defining $\gamma_{0}^{u1,FD}=2^{R_{1}}-1$ and $\gamma_{0}^{u2,FD}=2^{R_{2}}-1$, the outage probability of UE–1 can be obtained as*

(15) $${\mathrm{OP}}_{u1}^{FD}=\left\{\begin{array}{cc} 1, & \varpi_{1}^{FD}\leq 0\\ 1-\exp\left(\frac{-1}{\varpi_{1}^{FD}}\right){\left(\frac{\omega_{3}}{\varpi_{1}^{FD}}+1\right)}^{-1}, & \varpi_{1}^{FD}>0 \end{array}\right.$$

*where $\omega_{1}=\varepsilon_{1}\lambda_{\mathrm{bs},u1}$, $\omega_{2}=\varepsilon_{2}\lambda_{\mathrm{bs},u1}$, $\omega_{3}=\varepsilon_{3}\xi_{\mathrm{re},u1}\lambda_{\mathrm{re},u1}$, $\omega_{4}=\varepsilon_{4}\lambda_{\mathrm{bs},u1}$ and $\varpi_{1}^{FD}=\min\left(\frac{\omega_{4}}{\gamma_{0}^{u2,FD}}-\omega_{1},\frac{\omega_{1}}{\gamma_{0}^{u1,FD}}\right)-\omega_{2}$.*

**Proof.**  See Appendix B. □

#### 3.1.2. Outage Probability of UE–2 in FD Mode

**Proposition** **2.** *The outage probability of UE–2 can be derived as*

(16) $$\begin{array}{c} {\mathrm{OP}}_{u2}^{FD}=\left\{\begin{array}{cc} 1, & \varpi_{2}^{FD}\leq 0\\ 1-\frac{\upsilon_{1}^{FD}}{\varphi_{3}+\upsilon_{1}^{FD}}\exp\left(\frac{-1}{\upsilon_{1}^{FD}}-\frac{1}{\upsilon_{2}^{FD}}\right), & \varpi_{2}^{FD}>0 \end{array}\right. \end{array}$$

*where $\varphi_{1}=\phi_{1}\lambda_{\mathrm{bs},\mathrm{re}}$, $\varphi_{2}=\phi_{2}\lambda_{\mathrm{bs},\mathrm{re}}$, $\varphi_{3}=\phi_{3}\xi_{\mathrm{re},\mathrm{re}}\lambda_{\mathrm{re},\mathrm{re}}$, $\varphi_{4}=\phi_{4}\lambda_{\mathrm{re},u2}$, $\varphi_{5}=\phi_{5}\lambda_{\mathrm{re},u2}$, and $\varpi_{2}^{FD}=\min\left(\upsilon_{1}^{FD},\upsilon_{2}^{FD}\right)$ with $\upsilon_{1}^{FD}\overset{\Delta }{=}\frac{\varphi_{1}}{\gamma_{0}^{u2,FD}}-\varphi_{2}$, $\upsilon_{2}^{FD}\overset{\Delta }{=}\frac{\varphi_{4}}{\gamma_{0}^{u2,FD}}-\varphi_{5}$.*

**Proof** **2.** See Appendix C. □

#### 3.1.3. Overall System Outage Probability of FD Mode

The total outage probability of the considered FD cooperative NOMA network can be thus, obtained as

(17) $$\begin{array}{cc} {\mathrm{OP}}^{FD}= & \mathrm{Pr}\left(OP_{u1}^{FD}\cup OP_{u2}^{FD}\right)\\ = & 1-\mathrm{Pr}\left(\left(1-OP_{u1}^{FD}\right)\cap \left(1-OP_{u2}^{FD}\right)\right)\\ = & 1-\left(1-OP_{u1}^{FD}\right)\times \left(1-OP_{u2}^{FD}\right). \end{array}$$

### 3.2. Ergodic Capacity Analysis

In this section, we evaluate the ergodic sum rate of the cooperative NOMA scheme. The ergodic capacity of the system can be written as

(18) $$\begin{array}{cc} C & =E\left\{\log_{2}\left(1+\gamma \right)\right\}\\ & =\int_{0}^{\infty }\log_{2}\left(1+x\right)f_{\gamma }\left(x\right)dx\\ & =\frac{1}{\ln2}\int_{0}^{\infty }\frac{1-F_{\gamma }\left(x\right)}{1+x}dx. \end{array}$$

**Proposition** **3.** *The ergodic capacities of UE–1 and UE–2 are respectively given by*

(19) CUE−1FD=1ln2∫0ω1ω1ω2ω211+xexp−xω1−ω2x1+ω3xω1−ω2x−1dx

(20) $$\begin{array}{c} C_{\mathrm{UE}-2}^{FD}=\frac{1}{\ln2}\int_{0}^{\varpi_{3}}\frac{1}{1+x}\exp\left(\frac{-x}{\varphi_{4}-\varphi_{5}x}-\frac{x}{\omega_{4}-\left(\omega_{1}+\omega_{2}\right)x}-\frac{x}{\varphi_{1}-\varphi_{2}x}\right)\\ \times {\left(\frac{\omega_{3}x}{\omega_{4}-\left(\omega_{1}+\omega_{2}\right)x}+1\right)}^{-1}{\left(\frac{\varphi_{3}x}{\varphi_{1}-\varphi_{2}x}+1\right)}^{-1}dx \end{array}$$

*where ϖ3FD=minω4ω4ω1+ω2ω1+ω2,φ1φ1φ2φ2,φ4φ4φ5φ5 and the rest of the notations are defined in Propositions 1 and 2.*

**Proof** **3.** By using the proposed equation, we derived the ergodic sum rate of UE–1 and UE–2 as above and the proofs of aforementioned capacities are shown in the Appendix D. □

## 4. NOMA with Half-Duplex Cooperative Relaying System

In this section, we analyze the HD relaying NOMA system. Unlike the works in [33] which consider both two phase for UE–1 transmission. In this paper, we address only one first stage in UE–1 communication.

The SINRs in HD transmission mode need to be rewritten since SI and CCI is not present in HD transmission. For UE–1, the SINRs for UE–2 data and its own data decoding are given respectively by

(21) $$\gamma_{\mathrm{bs}\to u1}^{u2,HD}=\frac{\varepsilon_{4}{\left|h_{\mathrm{bs},u1}\right|}^{2}}{\left(\varepsilon_{1}+\varepsilon_{2}\right){\left|h_{\mathrm{bs},u1}\right|}^{2}+1},$$

(22) $$\gamma_{\mathrm{bs}\to u1}^{u1,HD}=\frac{\varepsilon_{1}{\left|h_{\mathrm{bs},u1}\right|}^{2}}{\varepsilon_{2}{\left|h_{\mathrm{bs},u1}\right|}^{2}+1}.$$

For UE–2, the SINRs of first and second hop are respectively given by

(23) $$\gamma_{\mathrm{bs}\to \mathrm{re}}^{u2,HD}=\frac{\phi_{1}{\left|h_{\mathrm{bs},\mathrm{re}}\right|}^{2}}{\phi_{2}{\left|h_{\mathrm{bs},\mathrm{re}}\right|}^{2}+1},$$

(24) $$\gamma_{\mathrm{re}\to u2}^{u2,HD}=\frac{{\phi_{4}}_{\mathrm{re}}{\left|h_{\mathrm{re},u2}\right|}^{2}}{\phi_{5}{\left|h_{\mathrm{re},u2}\right|}^{2}+1}.$$

It is worth to note that, HD system is no longer suffered by interferences (both SI at relay and CCI from relay to UE–1), however, the spectral efficiency gets reduced by the factor of 2, i.e., $C=0.5\log_{2}\left(1+\gamma \right)$.

### 4.1. Outage Probability Analysis for HD Mode

#### 4.1.1. Outage Probability of UE–1 for HD Network

Similar to the analysis done in FD system, the outage performance of UE–1 in HD transmission can be expressed as

(25) $$\begin{array}{cc} OP_{u1}^{HD}= & 1-\mathrm{Pr}\left(\gamma_{\mathrm{bs}\to u1}^{u2,HD}\geq \gamma_{0}^{u2,HD}\cap \gamma_{\mathrm{bs}\to u1}^{u1,HD}\geq \gamma_{0}^{u1,HD}\right)\\ = & \left\{\begin{array}{cc} 1, & \varpi_{1}^{HD}<0,\\ 1-\exp\left(-\frac{1}{\varpi_{1}^{HD}}\right), & \varpi_{1}^{HD}\geq 0, \end{array}\right. \end{array}$$

where $\varpi_{1}^{HD}=\min\left(\frac{\omega_{4}}{\gamma_{0}^{u2,HD}}-\omega_{1},\frac{\omega_{1}}{\gamma_{0}^{u1,HD}}\right)-\omega_{2}$.

#### 4.1.2. Outage Probability of UE–2 in HD Mode

The outage probability of UE–2 in the HD NOMA cooperative relaying scheme is evaluated as

(26) $$\begin{array}{cc} {\mathrm{OP}}_{u2}^{HD}= & 1-\mathrm{Pr}\left(\gamma_{\mathrm{bs}\to \mathrm{re}}^{u2,HD}\geq \gamma_{0}^{u2,HD}\cap \gamma_{\mathrm{re}\to u2}^{u2,HD}\geq \gamma_{0}^{u2,HD}\right)\\ = & 1-\mathrm{Pr}\left(\gamma_{\mathrm{bs}\to \mathrm{re}}^{u2,HD}\geq \gamma_{0}^{u2,HD}\right)\mathrm{Pr}\left(\gamma_{\mathrm{re}\to u2}^{u2,HD}\geq \gamma_{0}^{u2,HD}\right)\\ = & \left\{\begin{array}{cc} 1, & \varpi_{2}^{HD}\leq 0,\\ 1-\exp\left(\frac{-1}{\upsilon_{1}^{HD}}-\frac{1}{\upsilon_{2}^{HD}}\right), & \varpi_{2}^{HD}>0, \end{array}\right. \end{array}$$

where $\varpi_{2}^{HD}=\min\left(\upsilon_{1}^{HD},\upsilon_{2}^{HD}\right)$ with $\upsilon_{1}^{HD}\overset{\Delta }{=}\frac{\varphi_{1}}{\gamma_{0}^{u2,HD}}-\varphi_{2}$, $\upsilon_{2}^{HD}\overset{\Delta }{=}\frac{\varphi_{4}}{\gamma_{0}^{u2,HD}}-\varphi_{5}$.

#### 4.1.3. Overall System Outage Probability of HD Mode

The overall system outage probability in HD mode is given by

(27) $$\begin{array}{cc} {\mathrm{OP}}^{HD}= & \mathrm{Pr}\left(OP_{u1}^{HD}\cup OP_{u2}^{HD}\right)\\ = & 1-\mathrm{Pr}\left(\left(1-OP_{u1}^{HD}\right)\cap \left(1-OP_{u2}^{HD}\right)\right)\\ = & 1-\left(1-OP_{u1}^{HD}\right)\times \left(1-OP_{u2}^{HD}\right). \end{array}$$

### 4.2. Ergodic Capacity

Similar to the ergodic capacity analysis in FD mode, the capacities of UE–1 and UE–2, respectively, in HD transmission mode can be straightforwardly computed as

(28) CUE−1HD=12ln2∫0ω1ω1ω2ω211+xexp−xω1−ω2xdx,

(29) $$C_{\mathrm{UE}-2}^{FD}=\frac{1}{2\ln2}\int_{0}^{\varpi_{3}}\frac{1}{1+x}\exp\left(\frac{-x}{\varphi_{4}-\varphi_{5}x}-\frac{x}{\omega_{4}-\left(\omega_{1}+\omega_{2}\right)x}-\frac{x}{\varphi_{1}-\varphi_{2}x}\right)dx,$$

where symbols are defined as same those in previous section.

## 5. Adaptive Transmission Mode

In this section, we propose two schemes which can adaptively switch in order to enhance overall system outage performance. The first model, so called “Architecture I” (A−I), exploits FD and HD for cooperative relaying with NOMA network. The second scheme, called “Architecture II” (A−II), adaptively switches between NOMA and OMA (particularly orthogonal frequency duplexing multiple access—OFDMA) for FD cooperative relaying network.

### 5.1. Architecture 1: FD-HD Trade-Off for Cooperative NOMA Network

Let $\phi \overset{\Delta }{=}\left(\gamma_{\mathrm{bs}\to u1}^{u2}\geq \gamma_{0}^{u2}\cap \gamma_{\mathrm{bs}\to u1}^{u1}\geq \gamma_{0}^{u1}\cap \gamma_{\mathrm{bs}\to \mathrm{re}}^{u2}\geq \gamma_{0}^{u2}\right)$, then the selection criterion of suggested architecture 1 can be mathematically expressed as

(30) $$\mathrm{Mode}=\left\{\begin{array}{cc} \mathrm{FD}, & P\left(\phi \right)=1,\\ \mathrm{HD}, & P\left(\phi \right)=0. \end{array}\right.$$

#### 5.1.1. Outage Probability of UE–1 in A-I Scheme

(31) $$\begin{array}{cc} {\mathrm{OP}}_{u1}^{A-I}= & \mathrm{Pr}\left(\Omega_{u1}^{FD}:\mathrm{Mode}=\mathrm{FD}\right)\times \mathrm{Pr}\left(\mathrm{Mode}=\mathrm{FD}\right)\\ & +\mathrm{Pr}\left(\Omega_{u1}^{HD}:\mathrm{Mode}=\mathrm{HD}\right)\times \mathrm{Pr}\left(\mathrm{Mode}=\mathrm{HD}\right)\\ & \overset{\Delta }{=}OP_{u1-1}^{A-I}+OP_{u1-2}^{A-I}, \end{array}$$

where $\Omega_{u1}^{HD}\overset{\Delta }{=}\left(\gamma_{\mathrm{bs}\to u1}^{u2}<\gamma_{0}^{u2,\mathrm{FD}},\gamma_{\mathrm{bs}\to u1}^{u1}\geq \gamma_{0}^{u1,\mathrm{FD}}\right)$ and $\Omega_{u1}^{HD}\overset{\Delta }{=}\left(\gamma_{\mathrm{bs}\to u1}^{u2,HD}<\gamma_{0}^{u2,HD}\cup \gamma_{\mathrm{bs}\to u1}^{u1,HD}<\gamma_{0}^{u1,HD}\right)$.

**Proposition** **4.** *The UE–1 outage performance of proposed A−I can be written as*

(32) $${\mathrm{OP}}_{u1}^{A-I}=OP_{u1}^{HD}-\Psi_{\phi }+\Psi_{u1}^{A-I}$$

*where $\Psi_{\phi }\overset{\Delta }{=}\left(1-{\mathrm{OP}}_{u1}^{FD}\right){\mathrm{OP}}_{u2-1}^{FD}$ and*

$$\begin{array}{cc} \Psi_{u1}^{A-I}= & OP_{u2-1}\times \left[\exp\left(-\frac{1}{\varpi_{1}^{HD}}\right)\left(1-\exp\left(\frac{\varpi_{1}^{HD}-\varpi_{1}^{FD}}{\omega_{3}\varpi_{1}^{HD}}\right)\right)\right.\\ & \left.+\frac{\varpi_{1}^{FD}}{\omega_{3}+\varpi_{1}^{FD}}\exp\left(\frac{\varpi_{1}^{HD}-\omega_{3}-\varpi_{1}^{FD}}{\omega_{3}\varpi_{1}^{HD}}\right)\right], \end{array}$$

*with ${\mathrm{OP}}_{u1}^{FD}$ and ${\mathrm{OP}}_{u2-1}^{FD}$ are given in (15) and (16), respectively.*

**Proof** **4.** See Appendix E. □

#### 5.1.2. Outage Probability of UE–2 in A-I Scheme

The outage probability of UE–2 with architecture 1 implementation can be expressed as

(33) $$\begin{array}{cc} OP_{u2}^{A-I} & =\mathrm{Pr}\left(\Omega_{u2}^{FD}:\mathrm{Mode}=\mathrm{FD}\right)\times \mathrm{Pr}\left(\mathrm{Mode}=\mathrm{FD}\right)\\ & +\mathrm{Pr}\left(\Omega_{u2}^{HD}:\mathrm{Mode}=\mathrm{HD}\right)\times \mathrm{Pr}\left(\mathrm{Mode}=\mathrm{HD}\right), \end{array}$$

where $\Omega_{u2}^{FD}\overset{\Delta }{=}\gamma_{\mathrm{re}\to u2}^{u2}<\gamma_{0}^{u2}$, $\Omega_{u2}^{HD}\overset{\Delta }{=}\gamma_{\mathrm{bs}\to \mathrm{re}}^{u2,HD}<\gamma_{0}^{u2,HD}\cup \gamma_{\mathrm{re}\to u2}^{u2,HD}<\gamma_{0}^{u1,HD}$.

**Proposition** **5.** *The outage probability of UE–2 with A-I scheme is*

(34) $$OP_{u2}^{A-I}=OP_{u2-1}^{A-I}+OP_{u2-2}^{A-I}$$

*where $OP_{u2-1}^{A-I}$ and $OP_{u2-2}^{A-I}$ are given in (A24) and (A25) respectively.*

**Proof** **5.** See Appendix F. □

#### 5.1.3. Overall system Outage of A-I Scheme

The overall outage performance of A-I model is determined by the outage performances of UE–1 and UE–2, and is mathematically expressed as

(35) $$\begin{array}{cc} {\mathrm{OP}}^{A-I}\overset{\Delta }{=} & \mathrm{Pr}\left(\Omega_{u1}^{FD}\cup \Omega_{u2}^{FD}:\mathrm{Mode}=\mathrm{FD}\right)\times \mathrm{Pr}\left(\mathrm{Mode}=\mathrm{FD}\right)\\ & +\mathrm{Pr}\left(\Omega_{u1}^{HD}\cup \Omega_{u2}^{HD}:\mathrm{Mode}=\mathrm{HD}\right)\times \mathrm{Pr}\left(\mathrm{Mode}=\mathrm{HD}\right). \end{array}$$

**Proposition** **6.** *The overall system outage of A−I is given by*

(36) $$\begin{array}{cc} {\mathrm{OP}}^{A-I}= & OP_{u1-1}^{A-I}+OP_{u2-1}^{A-I}+{\mathrm{OP}}^{HD}-\Psi_{\phi }+\Psi_{u1-1}^{A-I}\Psi_{u2-1}^{A-I}\Psi_{u2-2}^{A-I}, \end{array}$$

*with ${\mathrm{OP}}^{HD}$, $\Psi_{u1-1}^{A-I}$, $\Psi_{u2-1}^{A-I}$ and $\Psi_{u2-2}^{A-I}$ are given in (27), (A23), (A27) and (A28) respectively.*

**Proof** **6.** The first part of right hand side in (35) is

(37) $$\begin{array}{cc} OP_{1}^{A-I}\overset{\Delta }{=} & \mathrm{Pr}\left(\left(\Omega_{u1}^{FD}\cup \Omega_{u2}^{FD}\right)\cap \phi \right)\\ = & OP_{u1-1}^{A-I}+OP_{u2-1}^{A-I}, \end{array}$$

and the second term is

(38) $$\begin{array}{cc} OP_{2}^{A-I}= & \mathrm{Pr}\left(\left(1-\gamma_{\mathrm{bs}\to u1}^{u2,HD}\geq \gamma_{0}^{u2,HD}\cap \gamma_{\mathrm{bs}\to u1}^{u1,HD}\geq \gamma_{0}^{u1,HD}\right.\right.\\ & \cap \left.\left.\gamma_{\mathrm{bs}\to \mathrm{re}}^{u2,HD}\geq \gamma_{0}^{u2,HD}\cap \gamma_{\mathrm{re}\to u2}^{u2,HD}\geq \gamma_{0}^{u1,HD}\right)\cap \left(1-\phi \right)\right)\\ = & 1-\mathrm{Pr}\left(\phi \right)-\mathrm{Pr}\left(\left(\gamma_{\mathrm{bs}\to u1}^{u2,HD}\geq \gamma_{0}^{u2,HD}\cap \gamma_{\mathrm{bs}\to u1}^{u1,HD}\geq \gamma_{0}^{u1,HD}\right)\right)\\ & \times \mathrm{Pr}\left(\left(\gamma_{\mathrm{bs}\to \mathrm{re}}^{u2,HD}\geq \gamma_{0}^{u2,HD}\cap \gamma_{\mathrm{re}\to u2}^{u2,HD}\geq \gamma_{0}^{u1,HD}\right)\right)\\ & +\mathrm{Pr}\left(\gamma_{\mathrm{bs}\to u1}^{u2,HD}\geq \gamma_{0}^{u2,HD}\cap \gamma_{\mathrm{bs}\to u1}^{u1,HD}\geq \gamma_{0}^{u1,HD}\right.\\ & \cap \left.\gamma_{\mathrm{bs}\to \mathrm{re}}^{u2,HD}\geq \gamma_{0}^{u2,HD}\cap \gamma_{\mathrm{re}\to u2}^{u2,HD}\geq \gamma_{0}^{u1,HD}\cap \phi \right)\\ = & {\mathrm{OP}}^{HD}-\Psi_{\phi }+\Psi_{u1-1}^{A-I}\Psi_{u2-1}^{A-I}\Psi_{u2-2}^{A-I}. \end{array}$$

This is end of proof. □

### 5.2. Architecture 2: NOMA-OMA Trade-Off for Cooperative FD Network

In this suggested scheme, the transmission mode is switched between FD NOMA relaying and FD OMA relaying based on the outage behavior. Firstly, we introduce the OMA cooperative relaying with time division multiple access in the following subsection.

#### 5.2.1. OMA FD Relaying Scheme

The SINR at UE–1 in OMA transmission strategy is

(39) $$\gamma_{\mathrm{bs}\to u1}^{u1,OMA}=\frac{\varepsilon_{1}^{OMA}{\left|h_{\mathrm{bs},u1}\right|}^{2}}{\varepsilon_{2}^{OMA}{\left|h_{\mathrm{bs},u1}\right|}^{2}+1}.$$

where $\varepsilon_{1}^{OMA}={\mathrm{SNR}}_{\mathrm{bs}}$ and $\varepsilon_{2}^{OMA}={\mathrm{SNR}}_{\mathrm{bs}}\left(\kappa_{\mathrm{bs}}^{2}+\vartheta_{u1}^{2}\right)$. The SINR for first and second hop of UE-2 are respectively given by

(40) $$\gamma_{\mathrm{bs}\to \mathrm{re}}^{u2,OMA}=\frac{\phi_{1}^{OMA}{\left|h_{\mathrm{bs},\mathrm{re}}\right|}^{2}}{\phi_{2}^{OMA}{\left|h_{\mathrm{bs},\mathrm{re}}\right|}^{2}+\phi_{3}{\left|{\hat{f}}_{\mathrm{re},\mathrm{re}}\right|}^{2}+1},$$

(41) $$\gamma_{\mathrm{re}\to u2}^{u2,OMA}=\frac{\phi_{4}{\left|h_{\mathrm{re},u2}\right|}^{2}}{\phi_{5}{\left|h_{\mathrm{re},u2}\right|}^{2}+1},$$

where $\phi_{1}^{OMA}={\mathrm{SNR}}_{\mathrm{bs}}$ and $\phi_{2}^{OMA}={\mathrm{SNR}}_{\mathrm{bs}}\left(\kappa_{\mathrm{bs}}^{2}+\vartheta_{\mathrm{re}}^{2}\right)$. Outage probability of UE–1 in OMA mode is given by

(42) $$OP_{u1}^{OMA}=\left\{\begin{array}{cc} 1, & \varpi_{1}^{OMA}<0,\\ 1-\exp\left(\frac{-1}{\varpi_{1}^{OMA}}\right), & \varpi_{1}^{OMA}\geq 0. \end{array}\right.$$

where $\gamma_{0}^{u1,\mathrm{OMA}}=2^{2R_{1}}-1$, $\varpi_{1}^{OMA}=\frac{\omega_{1}^{OMA}}{\gamma_{0}^{u1,OMA}}-\omega_{2}^{OMA}$, $\omega_{1}^{OMA}=\phi_{1}^{OMA}\lambda_{\mathrm{bs},u1}$ and $\omega_{2}^{OMA}=\phi_{1}^{OMA}\lambda_{\mathrm{bs},u1}$.

The outage probability of UE–2 for OMA transmission mode is

(43) $$OP_{u2}^{OMA}=\left\{\begin{array}{cc} 1, & \varpi_{2}^{OMA}<0,\\ 1-OP_{u2-1}^{OMA}\times OP_{u2-2}^{OMA}, & \varpi_{2}^{OMA}\geq 0. \end{array}\right.$$

where $\gamma_{0}^{u2,\mathrm{OMA}}=2^{2R_{2}}-1$, $OP_{u2-1}^{OMA}=\exp\left(\frac{-1}{\upsilon_{1}^{OMA}}\right)\times {\left(1+\frac{\varphi_{3}}{\upsilon_{1}^{OMA}}\right)}^{-1}$, $OP_{u2-2}^{OMA}=\exp\left(\frac{-1}{\upsilon_{2}^{OMA}}\right)$ and $\varpi_{2}^{OMA}=\min\left(\upsilon_{1}^{OMA},\upsilon_{2}^{OMA}\right)$ with $\upsilon_{1}^{OMA}\overset{\Delta }{=}\frac{\varphi_{1}^{OMA}}{\gamma_{0}^{u2,OMA}}-\varphi_{2}^{OMA}$, $\upsilon_{2}^{OMA}\overset{\Delta }{=}\frac{\varphi_{4}}{\gamma_{0}^{u2,OMA}}-\varphi_{5}$, $\varphi_{1}^{OMA}\;=\;\phi_{1}^{OMA}\lambda_{\mathrm{bs},u2}$ and $\varphi_{2}^{OMA}=\phi_{2}^{OMA}\lambda_{\mathrm{bs},u2}$.

According to the independence of channel coefficients, the overall system outage probability of OMA scheme can be derived by the same procedure of that of NOMA as below

(44) $$OP^{OMA}=1-\left(1-OP_{u1}^{OMA}\right)\left(1-OP_{u2}^{OMA}\right).$$

#### 5.2.2. Architecture II

In this subsection, we suggest the adaptive switch between NOMA and OMA transmission mode. The mode selection criterion of A−II scheme can be expressed as

(45) $$\mathrm{Mode}=\left\{\begin{array}{cc} \mathrm{NOMA}, & P\left(\phi \right)=1,\\ \mathrm{OMA}, & P\left(\phi \right)=0. \end{array}\right.$$

#### 5.2.3. Outage Probability of UE–1 in A-II Scheme

The outage of UE–1 in A−II model can be written as

(46) $$\begin{array}{cc} OP_{u1}^{A-II}= & \mathrm{Pr}\left(\Omega_{u1}^{FD}:\mathrm{Mode}=\mathrm{NOMA}\right)\times \mathrm{Pr}\left(\mathrm{Mode}=\mathrm{NOMA}\right)\\ & +\mathrm{Pr}\left(\Omega_{u1}^{OMA}:\mathrm{Mode}=\mathrm{OMA}\right)\times \mathrm{Pr}\left(\mathrm{Mode}=\mathrm{OMA}\right)\\ \overset{\Delta }{=} & OP_{u1-1}^{A-II}+OP_{u1-2}^{A-II}, \end{array}$$

where $\Omega_{u1}^{OMA}\overset{\Delta }{=}\gamma_{\mathrm{bs}\to u1}^{u1,OMA}<\gamma_{0}^{u1,OMA}$.

**Proposition** **7.** *The outage performance of UE–1 with architecture 2 is given as*

(47) $$OP_{u1}^{A-II}=OP_{u1}^{OMA}-\Psi_{\phi }+OP_{u2-1}\times \Psi_{u1-2}^{A-II},$$

*where*

(48) $$\Psi_{u1-2}^{A-II}=\left\{\begin{array}{cc} 0, & \min\left(\varpi_{1}^{OMA},\varpi_{1}\right)<0,\\ 1-OP_{u1}, & \varpi_{1}\leq \varpi_{1}^{OMA},\\ \Xi , & \varpi_{1}>\varpi_{1}^{OMA} \end{array}\right.$$

*with*

$$\begin{array}{cc} \Xi \overset{\Delta }{=} & \exp\left(-\frac{1}{\varpi_{1}^{OMA}}\right)\left(1-\exp\left(\frac{1}{\omega_{3}}-\frac{\varpi_{1}}{\omega_{3}\varpi_{1}^{OMA}}\right)\right)\\ & +\frac{\varpi_{1}}{\omega_{3}+\varpi_{1}}\exp\left(\frac{1}{\omega_{3}}-\frac{1}{\varpi_{1}^{OMA}}-\frac{\varpi_{1}}{\omega_{3}\varpi_{1}^{OMA}}\right). \end{array}$$

**Proof** **7.** In (46), the first component equal zero, i.e., ${\mathrm{OP}}_{u1-1}^{A-II}=0$, which can be obtained by the same stage in that of A−I model. In addition, the second element is

(49) $$\begin{array}{cc} OP_{u1-2}^{A-II}= & OP_{u1}^{OMA}-\mathrm{Pr}\left(\phi \right)+\mathrm{Pr}\left(\gamma_{\mathrm{bs}\to \mathrm{re}}^{u2}\geq \gamma_{0}^{u2}\right)\\ & \times \mathrm{Pr}\left(\gamma_{\mathrm{bs}\to u1}^{u1,OMA}\geq \gamma_{0}^{u1,OMA}\cap \gamma_{\mathrm{bs}\to u1}^{u2}\geq \gamma_{0}^{u2}\cap \gamma_{\mathrm{bs}\to u1}^{u1}\geq \gamma_{0}^{u1}\right)\\ = & OP_{u1}^{OMA}-\Psi_{\phi }+OP_{u2-1}^{FD}\times \Psi_{u1-2}^{A-II}. \end{array}$$

Similar to the analysis done in architecture 1, we can obtain $\Psi_{u1-2}^{A-II}$. Thus, the proposition 7 is revealed. □

#### 5.2.4. Outage Probability of U–2 in A-II Scheme

The block probability of UE–2 in A−II can be mathematically formulated by

(50) $$\begin{array}{cc} {\mathrm{OP}}_{u2}^{A-II}= & \mathrm{Pr}\left(\Omega_{u2}^{FD}:\mathrm{Mode}=\mathrm{NOMA}\right)\times \mathrm{Pr}\left(\mathrm{Mode}=\mathrm{NOMA}\right)\\ & +\mathrm{Pr}\left(\Omega_{u2}^{OMA}:\mathrm{Mode}=\mathrm{OMA}\right)\times \mathrm{Pr}\left(\mathrm{Mode}=\mathrm{OMA}\right), \end{array}$$

where $\Omega_{u2}^{OMA}\overset{\Delta }{=}\gamma_{\mathrm{bs}\to \mathrm{re}}^{u2,OMA}<\gamma_{0}^{u2,OMA}\cup \gamma_{\mathrm{re}\to u2}^{u2,OMA}<\gamma_{0}^{u1,OMA}$.

**Proposition** **8.** *The block performance of UE–2 with proposed A−II scheme is*

(51) $${\mathrm{OP}}_{u2}^{A-II}=OP_{u2-1}^{A-I}+OP_{u2}^{OMA}-\Psi_{\phi }+\Psi_{u2}^{A-II},$$

*with $\Psi_{u2}^{A-II}$ is given in (55) and other terms are given in corresponding previous sections.*

**Proof** **8.** The first part in (50) can be obtained as

(52) $$OP_{u2-1}^{A-II}=OP_{u2-1}^{A-I}.$$

The second term in (50) is determined by similar steps of that in A-I as

(53) $$OP_{u2-2}^{A-II}=OP_{u2}^{OMA}-\Psi_{\phi }+\Psi_{u2}^{A-II}.$$

The last term in (53) can be determined as

(54) $$\begin{array}{cc} \Psi_{u2}^{A-II}\overset{\Delta }{=} & \mathrm{Pr}\left(\gamma_{\mathrm{re}\to u2}^{u2,OMA}\geq \gamma_{0}^{u1,OMA}\right)\\ & \times \mathrm{Pr}\left(\gamma_{\mathrm{bs}\to u1}^{u2}\geq \gamma_{0}^{u2}\cap \gamma_{\mathrm{bs}\to u1}^{u1}\geq \gamma_{0}^{u1}\right)\\ & \times \mathrm{Pr}\left(\gamma_{\mathrm{bs}\to \mathrm{re}}^{u2,OMA}\geq \gamma_{0}^{u2,OMA}\cap \gamma_{\mathrm{bs}\to \mathrm{re}}^{u2}\geq \gamma_{0}^{u2}\right)\\ = & OP_{u2-2}^{OMA}\times \left(1-OP_{u1}^{FD}\right)\times \Psi_{u2-1}^{A-II}. \end{array}$$

with

(55) $$\begin{array}{cc} \Psi_{u2-1}^{A-II}= & \mathrm{Pr}\left(\frac{\upsilon_{\min}}{\lambda_{\mathrm{bs},\mathrm{re}}}{\left|h_{\mathrm{bs},\mathrm{re}}\right|}^{2}\geq \phi_{3}{\left|{\hat{f}}_{\mathrm{re},\mathrm{re}}\right|}^{2}+1\right)\\ = & \left\{\begin{array}{cc} 0, & \upsilon_{\min}\leq 0,\\ \frac{\upsilon_{\min}}{\varphi_{3}+\upsilon_{\min}}\exp\left(\frac{-1}{\upsilon_{\min}}\right), & \upsilon_{\min}>0. \end{array}\right. \end{array}$$

where $\upsilon_{\min}\overset{\Delta }{=}\min\left(\upsilon_{1}^{OMA},\upsilon_{1}^{FD}\right)$. □

#### 5.2.5. Overall system Outage of A-II scheme

**Proposition** **9.** *The overall outage performance of mode selection scheme 2 can be expressed as*

(56) $$OP^{A-II}=OP_{1}^{A-II}+OP_{2}^{A-II}$$

*where*

(57) $$\begin{array}{cc} OP_{1}^{A-II}\overset{\Delta }{=} & \mathrm{Pr}\left(\left(\Omega_{u1}\cup \Omega_{u2}\right)\cap \phi \right)\\ = & OP_{u1-1}^{A-II}+OP_{u2-1}^{A-II}, \end{array}$$

*and*

(58) $$OP_{2}^{A-II}=OP^{OMA}-\Psi_{\phi }+OP_{u2-2}^{OMA}\Psi_{u1-2}^{A-II}\Psi_{u2-1}^{A-II}.$$

**Proof** **9.** The result can be obtained by doing the similar analysis done in proposition 6. □

## 6. Simulation Results and Discussions

In this section, Monte Carlo simulations are presented to corroborate the theoretical results. In particular, considering the aforementioned FD or HD scenarios in NOMA approach, this section investigates the effect of hardware impairments on the performance of NOMA over Rayleigh fading conditions. The simulation results are obtained by running simulations in random channel realizations. Furthermore, for a reasonable comparison, we assume that the transmit power level is always fixed. For both FD/HD and OMA/NOMA adaptive architectures, it is observed that the derived outage probability and ergodic capacity perfectly matches with the simulations over entire range of SNR and other parameters which include power allocation factor, hardware impairment levels. In this comment correspondence, the parameters are chosen similar to that in [34]. In particular, ${SNR}_{\mathrm{re}}=0.5{SNR}_{\mathrm{bs}}$, $a_{1}=0.15$, $a_{2}=0.85$, $\lambda_{\mathrm{bs},u1}=0.8$, $\lambda_{\mathrm{bs},\mathrm{re}}=\lambda_{\mathrm{re},u1}=\lambda_{\mathrm{re},u2}=0.5$, $\lambda_{\mathrm{re},\mathrm{re}}=1$, $\xi_{\mathrm{re},u1}=\xi_{\mathrm{re},\mathrm{re}}=0.01$. We also assume $\kappa_{\mathrm{bs}}^{2}=\kappa_{\mathrm{re}}^{2}\overset{\Delta }{=}\kappa$, $\vartheta_{\mathrm{re}}^{2}=\vartheta_{u1}^{2}=\vartheta_{u2}^{2}\overset{\Delta }{=}\vartheta$ and κ=ϑ. Without the loss of generality, we set $\xi_{\mathrm{re},\mathrm{re}}=\xi_{\mathrm{re},u1}=0.01$, $\kappa^{2}=\vartheta^{2}=0.01$.

### 6.1. Ergodic Capacity Examinations

**Figure 3:**
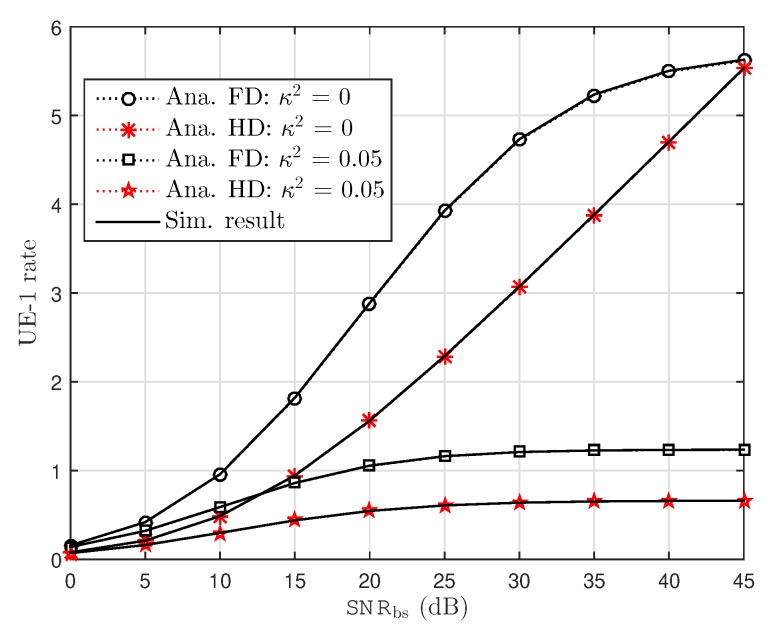
UE–1 capacity versus BS transmission ${SNR}_{\mathrm{bs}}$ with several value of RHI.

**Figure 4:**
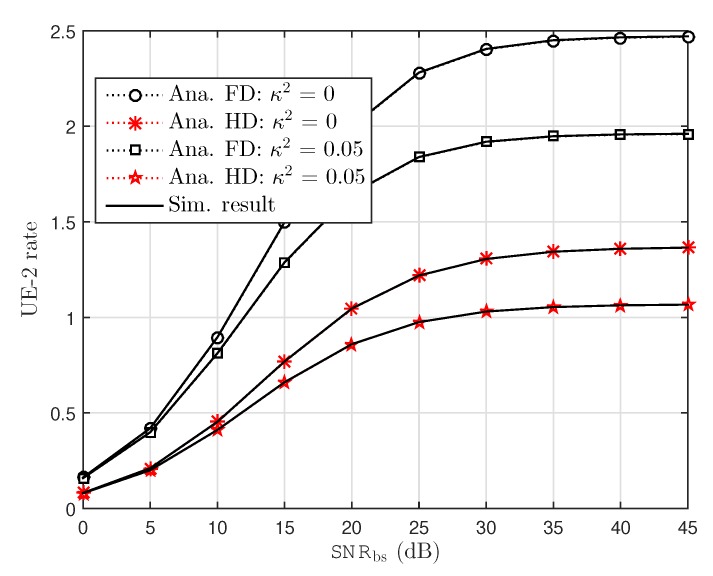
UE–2 capacity versus BS transmission ${SNR}_{\mathrm{bs}}$ with several value of RHI.

In Figure 3, the rate performance at UE–1 under impact of RHI noise in FD and HD schemes is considered as a function related to SNR at source. Herein, two different values of RHI levels: $\kappa^{2}\in \left[0.05,0\right]$ are plotted to show the performance gap. from the results, one can clearly observe and confirm that rate performance for FD is higher than HD case. From Figure 3, it can be seen that the analytical results exactly matches with the simulation results over the entire range of SNR, and the performance gap at different levels of RHI can be seen clearly at high SNRs. Additionally, it can be confirmed that NOMA with the ideal hardware reaches maximal performance than the other case. It is noted that rate of UE–1 remains constant as SNR is increased beyond 30 dB under impact of concerned level of RHI. However, power allocation factor is set at a different value for UE–2 as compared to UE–1 and in such situation results in lower rate as observation in Figure 4. The ceiling rate at UE–2 can be obtained at two concerned levels of RHI. This illustration shows limitation of maximal rate in UE–2 at high SNR.

**Figure 5:**
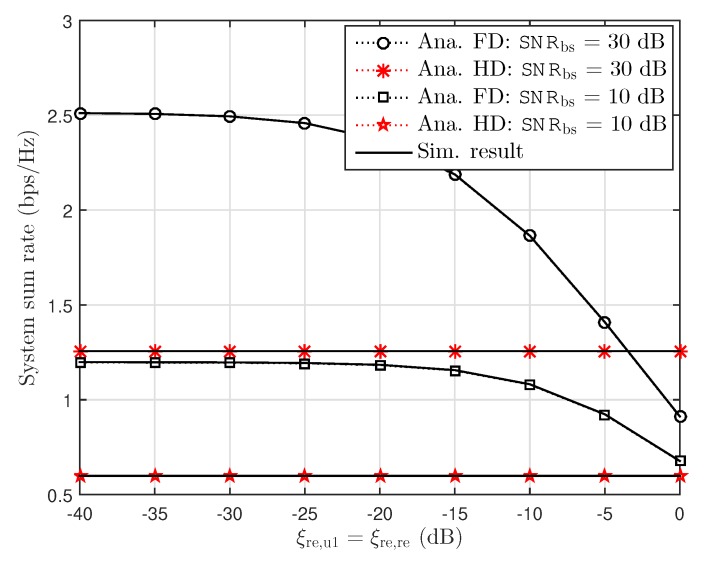
Sum rate versus interference cancellation level with $\xi_{\mathrm{re},\mathrm{re}}=\xi_{\mathrm{re},u1}$, $\kappa^{2}=\vartheta^{2}=0.01$ and ${SNR}_{\mathrm{bs}}=\left[10,30\right]$ dB.

It is pointed out in Figure 5 that higher SNR at BS leads to higher system sum rate as considerding residual interference due to FD. Such performance in terms of system sum rate declines with an increase in residual interference. It is also clear from Figure 5 that the sum rate of the system is straight line at HD mode due to the absence of residual interference.

**Figure 6:**
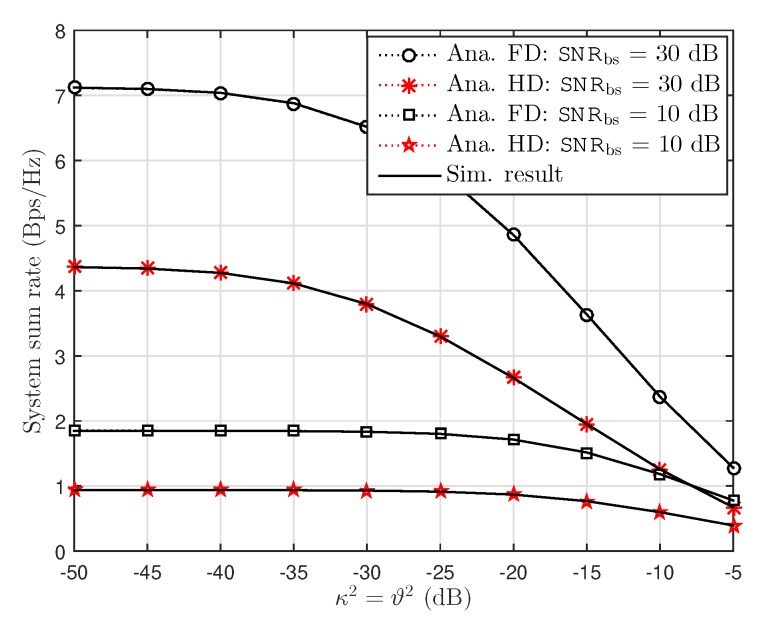
Sum rate versus RHI level with $\kappa^{2}=\vartheta^{2}$, $\xi_{\mathrm{re},\mathrm{re}}=\xi_{\mathrm{re},u1}=0.01$ and ${SNR}_{\mathrm{bs}}=\left[10,30\right]$ dB.

Figure 6 examines the effect of level of RHI $\kappa^{2}=\vartheta^{2}$ on system sum rate. It can be seen clearly that higher level of noise due to imperfection of hardware is main reason to show decreasing sum rate. At small amount of RHI, the ceiling sum rate can be observed, however, sum rate is small as $\kappa^{2}=\vartheta^{2}$ is greater than –10 (dB). In this situation, FD performance is better than HD at dedicated SNR at the BS.

### 6.2. Outage Probability Examinations

**Figure 7:**
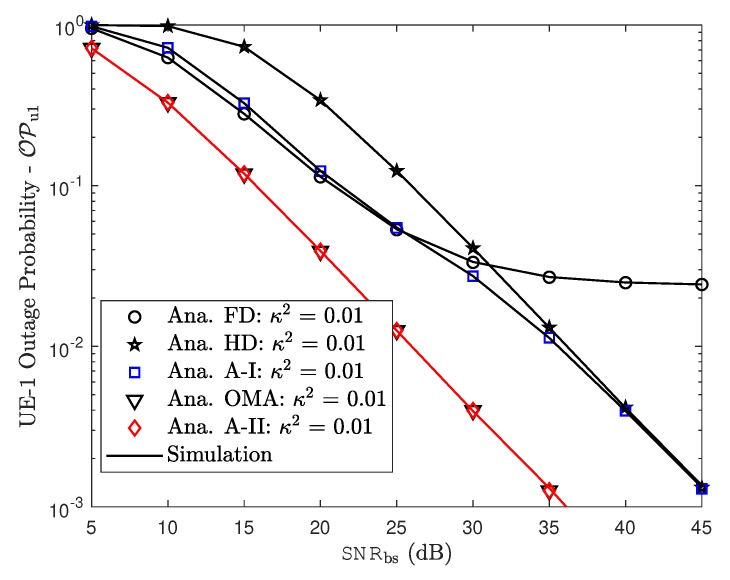
Outage probability of UE–1.

**Figure 8:**
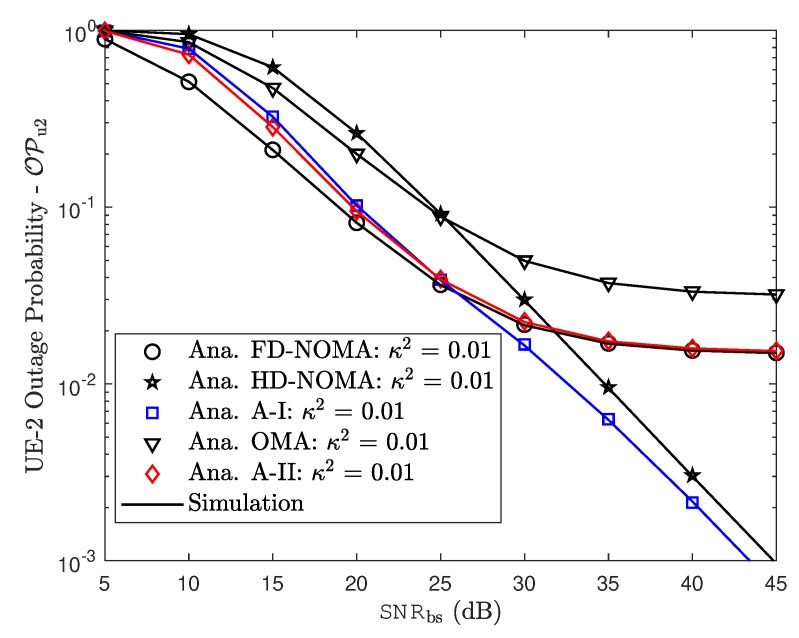
Outage probability of UE–2.

Figure 7 and Figure 8 demonstrate the impact of hardware impairment on outage performance for both users in NOMA as considering tradeoff between OMA/NOMA cases and FD/HD cases. From these experimental results, it can be observed that, the proposed two adaptive schemes provide improved outage performance. The outage floors for FD mode at both NOMA users at high SNR are also provided. This observation is consistent with derived formula. In such case, RHI contribute to degrade system performance in two NOMA users and it is proper trend with other simulation results.

**Figure 9:**
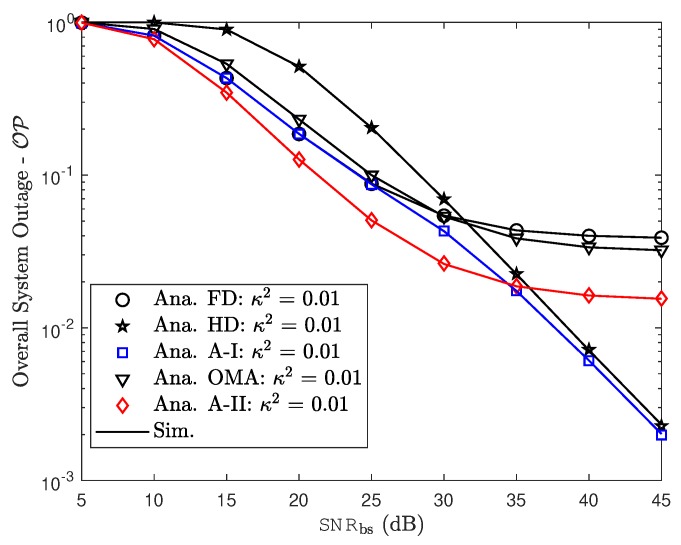
Overall system outage probability as a function of transmit power ${\mathrm{SNR}}_{\mathrm{bs}}$.

**Figure 10:**
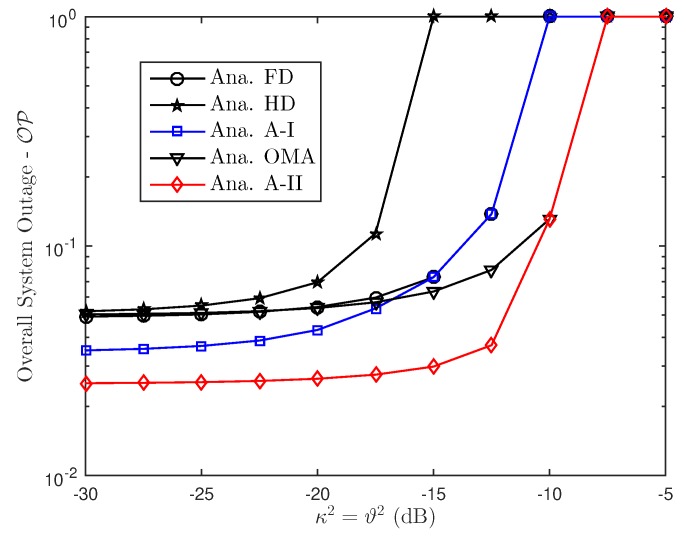
Overall system outage probability versus RHI.

**Figure 11:**
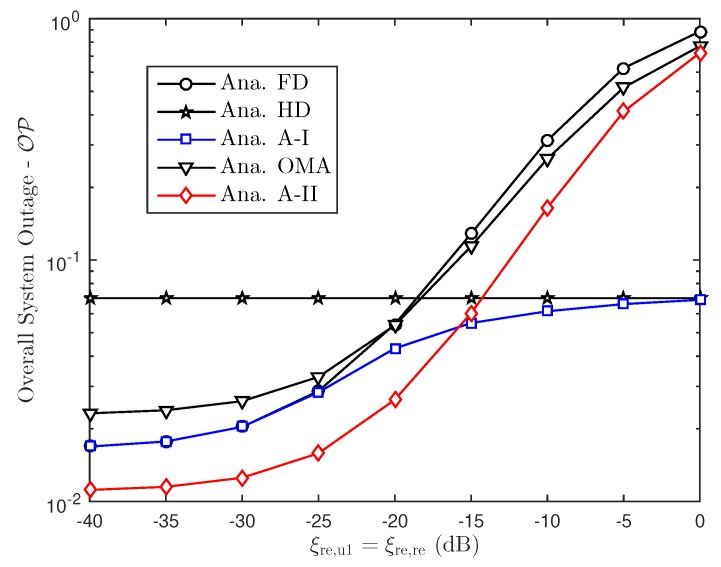
Overall system outage probability versus residual interference cancellation level.

To analyze the proposed scheme, we perform simulation as in Figure 9 for overall outage performance. Comparison study related to outage performance in this situation provide advantage of adaptive scheme. In addition, it can be observed that the overall outage performance varies with the residual interference because of FD mode and RHI levels as illustrated in Figure 10 and Figure 11.

**Figure 12:**
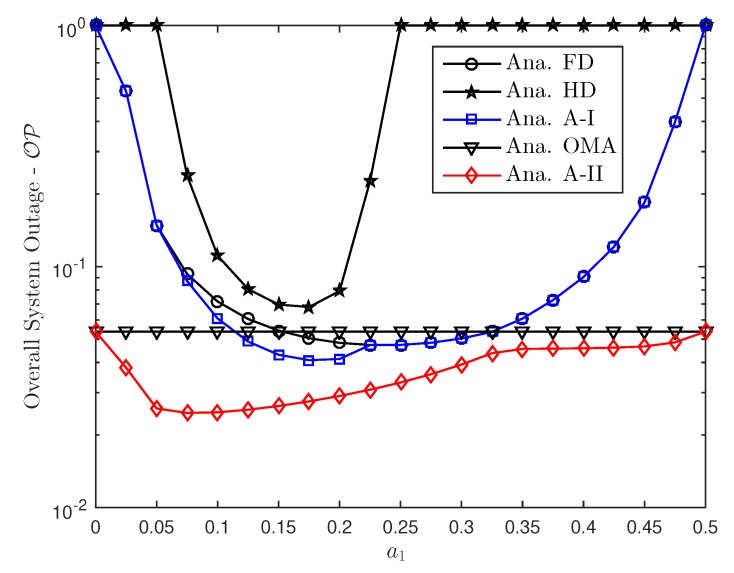
Overall system outage probability curve as a function of power allocation $a_{1}$.

Figure 12 studies the overall outage performance of considered NOMA system as a function of power allocation. It is noted that such optimal power allocation factors can be obtained in numerical method to highlight optimal performance. The significant fluctuation can be seen in HD NOMA mode as is greater than 0.25 the outage event occurred. The power allocation factors affect all the schemes in term of overall outage performance. This will be become an important aspect in the design of NOMA if it can be controlled level of allocated power for each user.

## 7. Conclusions

In this paper, we analyzed a cooperative FD NOMA transmission scheme considering the fact that some users in NOMA systems may need a relay node to forward a signal to a far user. The relay node is assumed to have imperfect hardware (hardware impairments). It is observed that the residual interference resulted from FD and RHI from non-ideal hardware can severely degrade the system performance. The adaptive schemes are introduced to achieve an advantage of FD and NOMA compared with a traditional HD OMA system. Analytical results have been derived to observe the outage and ergodic capacity performances of the concerned schemes. A numerical method is applied to find optimal power allocation quantities for two NOMA users for optimal performance.

## Appendix A. Lemma 1

The CDF of *Z* can be calculated as

(A1) FZt=PrZ≤t=PraXbX+cY+1≤t=Praatt−bX≤cY+1.

We now consider two scenarios discussed below. In the case of t>aabb, the probability in (A1) is always equal to one, $F_{Z}\left(t\right)=1$, caused this scenario lead to a probability of negative number smaller than a number at least equal to 1. Otherwise, when t≤aabb the probability can be determined as

(A2) FZt=∫0∞FX−cy+1aatt−bfY−ydy=1−1λY∫0∞exp−cy+1aatt−bλXexp−yλYdy=1−exp−ta−btλX1+ctλYa−btλX−1.

Combining two cases above, the Appendix A is derived, this is end of explanation.

## Appendix B. Proof of Proposition 1

Substituting (4) and (6) into (14) and after some simple mathematical manipulation, the outage probability at UE–1 can be simplified as

(A3) $$\begin{array}{c} {\mathrm{OP}}_{u1}^{FD}=1-\mathrm{Pr}\left\{\frac{{\left|h_{\mathrm{bs},u1}\right|}^{2}\left(\frac{\varepsilon_{4}}{\gamma_{0}^{u2}}-\varepsilon_{1}-\varepsilon_{2}\right)}{\varepsilon_{3}{\left|{\hat{f}}_{\mathrm{re},u1}\right|}^{2}+1}\geq 1\right.\cap \left.\frac{{\left|h_{\mathrm{bs},u1}\right|}^{2}\left(\frac{\varepsilon_{1}}{\gamma_{0}^{u1}}-\varepsilon_{2}\right)}{\varepsilon_{3}{\left|{\hat{f}}_{\mathrm{re},u1}\right|}^{2}+1}\geq 1\right\}. \end{array}$$

We consider two scenarios as below.

*Case 1:* when a2a2γ0u2γ0u2−a1<a1a1γ0u1γ0u1, the outage probability gets reduced to

(A4) $$\begin{array}{c} {\mathrm{OP}}_{u1}^{FD}=1-\mathrm{Pr}\left\{{\left|h_{\mathrm{bs},u1}\right|}^{2}\left(\frac{\varepsilon_{4}}{\gamma_{0}^{u2}}-\varepsilon_{1}-\varepsilon_{2}\right)\right.\left.\geq \varepsilon_{3}{\left|{\hat{f}}_{\mathrm{re},u1}\right|}^{2}+1\right\} \end{array}$$

*Case 2:* when $a_{2}/\gamma_{0}^{u2}-a_{1}\geq a_{1}/\gamma_{0}^{u1}$ the outage probability can be written as

(A5) $$\begin{array}{c} {\mathrm{OP}}_{u1}^{FD}=1-\mathrm{Pr}\left\{{\left|h_{\mathrm{bs},u1}\right|}^{2}\left(\frac{\varepsilon_{1}}{\gamma_{0}^{u1}}-\varepsilon_{2}\right)\right.\left.\geq \varepsilon_{3}{\left|{\hat{f}}_{\mathrm{re},u1}\right|}^{2}+1\right\} \end{array}$$

With the help of Appendix A, the problem in (A4) and (A5) are easily obtained. Recalling *Case 1* and *Case 2*, the proposition 1 is derived, this is end.

## Appendix C. Proof of Proposition 2

The outage probability of UE–2 can be expressed as

(A6) $$OP_{u2}^{FD}=1-\mathrm{Pr}\left\{\min\left(\gamma_{\mathrm{re}\to u2}^{u2},\gamma_{\mathrm{bs}\to \mathrm{re}}^{u2}\right)>\gamma_{0}^{u2}\right\}.$$

Since $\gamma_{\mathrm{re}\to u2}^{u2}$ and $\gamma_{\mathrm{bs}\to \mathrm{re}}^{u2}$ are independence to each other, $OP_{u2}$ can be further simplified as

(A7) $$\begin{array}{cc} {\mathrm{OP}}_{u2}^{FD}= & 1-\underset{OP_{u2-1}^{FD}}{\underset{︸}{\mathrm{Pr}\left\{\gamma_{\mathrm{bs}\to \mathrm{re}}^{u2}>\gamma_{0}^{u2}\right\}}}\times \underset{OP_{u2-2}^{FD}}{\underset{︸}{\mathrm{Pr}\left\{\gamma_{\mathrm{re}\to u2}^{u2}>\gamma_{0}^{u2}\right\}}}. \end{array}$$

substituting (9) into (A7), the first term in above equation can be expressed as

(A8) $${\mathrm{OP}}_{u2-1}^{FD}=\mathrm{Pr}\left(\frac{\phi_{1}{\left|h_{\mathrm{bs},\mathrm{re}}\right|}^{2}}{\phi_{2}{\left|h_{\mathrm{bs},\mathrm{re}}\right|}^{2}+\phi_{3}{\left|{\hat{f}}_{\mathrm{re},\mathrm{re}}\right|}^{2}+1}\geq \gamma_{0}^{u2}\right).$$

If this condition $\frac{a_{2}}{a_{1}+\kappa_{\mathrm{bs}}^{2}+\vartheta_{\mathrm{re}}^{2}}<\gamma_{0}^{u2}$ is satisfied, then $OP_{u2-1}=0$. In the event of $\frac{a_{2}}{a_{1}+\kappa_{\mathrm{bs}}^{2}+\vartheta_{\mathrm{re}}^{2}}\geq \gamma_{0}^{u2}$, the first part of UE–2 outage is given as above with the help of Appendix A.

In particular, we set $\varphi_{1}=\phi_{1}\lambda_{\mathrm{bs},\mathrm{re}}$, $\varphi_{2}=\phi_{2}\lambda_{\mathrm{bs},\mathrm{re}}$, $\varphi_{3}=\phi_{3}\xi_{\mathrm{re},\mathrm{re}}\lambda_{\mathrm{re},\mathrm{re}}$, $OP_{u2-1}$ is calculated as below

(A9) $$OP_{u2-1}^{FD}=\exp\left(\frac{-1}{\upsilon_{1}^{FD}}\right){\left(1+\frac{\varphi_{3}}{\upsilon_{1}^{FD}}\right)}^{-1}.$$

In addition, the second item in (A7) can be calculated as

(A10) $$OP_{u2-2}^{FD}=\mathrm{Pr}\left({\left|h_{\mathrm{re},u2}\right|}^{2}\left(\frac{\phi_{4}}{\gamma_{0}^{u2}}-\phi_{5}\right)\geq \gamma_{0}^{u2}\right).$$

If $\frac{1}{\kappa_{\mathrm{re}}^{2}+\vartheta_{u2}^{2}}<\gamma_{0}^{u2}$ then $OP_{u2-2}=0$. Otherwise, if $\frac{1}{\kappa_{\mathrm{re}}^{2}+\vartheta_{u2}^{2}}\geq \gamma_{0}^{u2}$ then

(A11) $${\mathrm{OP}}_{u2-2}^{FD}=\exp\left(\frac{-1}{\upsilon_{2}^{FD}}\right)$$

Finally, the proposition 2 is derived.

## Appendix D. Proof of Proposition 3

### Appendix D.1. Ergodic Capacity of UE–1

The Ergodic sum rate of UE–1 can be written as

(A12) $$\begin{array}{cc} C_{\mathrm{UE}-1} & =E\left\{\log_{2}\left(1+\gamma_{\mathrm{bs}\to u1}^{u1}\right)\right\}\\ & =\frac{1}{\ln2}\int_{0}^{\infty }\frac{1}{1+x}\left(1-F_{\gamma_{\mathrm{bs}\to u1}^{u1}}\left(x\right)\right)dx \end{array}$$

With the help of Appendix A, this first part can get the CDF of $\gamma_{\mathrm{bs}\to u1}^{u1}$, $F_{\gamma_{\mathrm{bs}\to u1}^{u1}}\left(x\right)$ as below

(A13) Fγbs→u1u1x=Prγbs→u1u1<x=1,t>ω1ω1ω2ω2,1−exp−xω1−ω2x1+ω3xω1−ω2x−1,t≤ω1ω1ω2ω2,

where $\omega_{1},\omega_{2},\omega_{3}$ are defined in Proposition 1. Substituting (A13) into (A12) we can obtain $C_{\mathrm{UE}-1}$ as

(A14) CUE−1=1ln2∫0ω1ω1ω2ω211+xexp−xω1−ω2x1+ω3xω1−ω2x−1dx.

### Appendix D.2. Ergodic Capacity of UE–2

We set $\gamma_{e2e}^{u2}=\min\left\{\gamma_{\mathrm{bs}\to u1}^{u2},\gamma_{\mathrm{bs}\to \mathrm{re}}^{u2},\gamma_{\mathrm{re}\to u2}^{u2}\right\}$, then the achievable data rate of UE–2 can be obtained as

(A15) $$\begin{array}{cc} E\left\{C_{\mathrm{UE}-2}\right\} & =E\left\{\log_{2}\left(1+\min\left\{\gamma_{\mathrm{bs}\to u1}^{u2},\gamma_{\mathrm{bs}\to \mathrm{re}}^{u2},\gamma_{\mathrm{re}\to u2}^{u2}\right\}\right)\right\}\\ & =\frac{1}{\ln2}\int_{0}^{\infty }\frac{1}{1+x}\left(1-F_{\gamma_{e2e}^{u2}}\left(x\right)\right)dx \end{array}$$

where $F_{\gamma_{e2e}^{u2}}\left(x\right)=1-\mathrm{Pr}\left\{\gamma_{\mathrm{bs}\to u1}^{u2}\geq x\right\}\mathrm{Pr}\left\{\gamma_{\mathrm{bs}\to \mathrm{re}}^{u2}\geq x\right\}\mathrm{Pr}\left\{\gamma_{\mathrm{re}\to u2}^{u2}\geq x\right\}$.

The CDF of $\gamma_{e2e}^{u2}$ can be calculated as following

(A16) $$\begin{array}{cc} F_{\gamma_{e2e}^{u2}}\left(x\right) & =\mathrm{Pr}\left\{\min\left\{\gamma_{\mathrm{bs}\to u1}^{u2},\gamma_{\mathrm{bs}\to \mathrm{re}}^{u2},\gamma_{\mathrm{re}\to u2}^{u2}\right\}<x\right\}\\ & =1-\mathrm{Pr}\left\{\gamma_{\mathrm{bs}\to u1}^{u2}\geq x\right\}\mathrm{Pr}\left\{\gamma_{\mathrm{bs}\to \mathrm{re}}^{u2}\geq x\right\}\mathrm{Pr}\left\{\gamma_{\mathrm{re}\to u2}^{u2}\geq x\right\}. \end{array}$$

After some mathematical manipulations, the following conditions can be obtained with the help of Appendix A as

(A17) Prγbs→u1u2≥x=0,x>ω4ω4ω1+ω2ω1+ω2exp−xω4−ω1+ω2x×ω3xω4−ω1+ω2x+1−1,x≤ω4ω4ω1+ω2ω1+ω2.

(A18) Prγbs→reu2≥x=0,x>φ1φ1φ2φ2exp−xφ1−φ2xφ3xφ1−φ2x+1−1,x≤φ1φ1φ2φ2.

(A19) Prγre→u2u2≥x=0,x>φ4φ4φ5φ5exp−xφ4−φ5x,x≤φ4φ4φ5φ5.

Replacing (A17) (A18) and (A19) into (A16) and with the help of Appendix A, the sum rate of UE–2 can be easily obtained. The parameters $\omega_{1}$$\omega_{2}$$\omega_{3}$$\omega_{4}$$\varphi_{4}$ and $\varphi_{5}$ are defined in Propositions 1 and 2.

## Appendix E. Proof of Proposition 4

It is noted that, the first term of right hand side in (31) is zero, i.e., $OP_{u1-1}^{A-I}=0$. Thus, the main task now is determine the second term. With the equality of a condition probability $\mathrm{Pr}\left(A:B\right)=\mathrm{Pr}\left(A,B\right)/\mathrm{Pr}\left(B\right)$, Equation (31) can be rewritten after some manipulations as

(A20) $$\begin{array}{cc} OP_{u1-2}^{A-I}= & 1-\mathrm{Pr}\left(\gamma_{\mathrm{bs}\to u1}^{u2,HD}\geq \gamma_{0}^{u2,HD}\cap \gamma_{\mathrm{bs}\to u1}^{u1,HD}\geq \gamma_{0}^{u1,HD}\right)\\ & -\mathrm{Pr}\left(\phi \right)+\mathrm{Pr}\left(\gamma_{\mathrm{bs}\to u1}^{u2,HD}\geq \gamma_{0}^{u2,HD}\cap \gamma_{\mathrm{bs}\to u1}^{u1,HD}\geq \gamma_{0}^{u1,HD}\cap \phi \right)\\ = & OP_{u1}^{HD}-\mathrm{Pr}\left(\phi \right)+\mathrm{Pr}\left(\gamma_{\mathrm{bs}\to u1}^{u2,HD}\geq \gamma_{0}^{u2,HD}\cap \gamma_{\mathrm{bs}\to u1}^{u1,HD}\geq \gamma_{0}^{u1,HD}\cap \phi \right). \end{array}$$

The second component in (A20) is given by

(A21) $$\begin{array}{cc} \Psi_{\phi }\overset{\Delta }{=} & \mathrm{Pr}\left(\phi \right)=\mathrm{Pr}\left(\gamma_{\mathrm{bs}\to u1}^{u2}\geq \gamma_{0}^{u2}\cap \gamma_{\mathrm{bs}\to \mathrm{re}}^{u2}\geq \gamma_{0}^{u2}\cap \gamma_{\mathrm{bs}\to u1}^{u1}\geq \gamma_{0}^{u1}\right)\\ = & \mathrm{Pr}\left(\gamma_{\mathrm{bs}\to u1}^{u2}\geq \gamma_{0}^{u2}\cap \gamma_{\mathrm{bs}\to \mathrm{re}}^{u2}\geq \gamma_{0}^{u2}\right)\mathrm{Pr}\left(\gamma_{\mathrm{bs}\to u1}^{u1}\geq \gamma_{0}^{u1}\right)\\ = & \left(1-OP_{u1}^{FD}\right)OP_{u2-1}^{FD}. \end{array}$$

Substituting into the last element in (A20), we get

(A22) $$\begin{array}{cc} \Psi_{u1}^{A-I}= & \mathrm{Pr}\left(\gamma_{\mathrm{bs}\to u1}^{u2,HD}\geq \gamma_{0}^{u2,HD}\cap \gamma_{\mathrm{bs}\to u1}^{u1,HD}\geq \gamma_{0}^{u1,HD}\right.\\ & \left.\cap \gamma_{\mathrm{bs}\to u1}^{u2}\geq \gamma_{0}^{u2}\cap \gamma_{\mathrm{bs}\to u1}^{u1}\geq \gamma_{0}^{u1}\right)\times \mathrm{Pr}\left(\gamma_{\mathrm{bs}\to \mathrm{re}}^{u2}\geq \gamma_{0}^{u2}\right)\\ \overset{\Delta }{=} & \Psi_{u1-1}^{A-I}\times \Psi_{u1-2}^{A-I}, \end{array}$$

with

(A23) $$\begin{array}{cc} \Psi_{u1-1}^{A-I}= & \mathrm{Pr}\left({\left|h_{\mathrm{bs},u1}\right|}^{2}\geq \frac{\lambda_{\mathrm{bs},u1}}{\varpi_{1}^{HD}}\cap {\left|h_{\mathrm{bs},u1}\right|}^{2}\geq \frac{\varepsilon_{3}\lambda_{\mathrm{bs},u1}{\left|{\hat{f}}_{\mathrm{re},u1}\right|}^{2}}{\varpi_{1}}+\frac{\lambda_{\mathrm{bs},u1}}{\varpi_{1}}\right)\\ = & \exp\left(-\frac{1}{\varpi_{1}^{HD}}\right)\left(1-\exp\left(\frac{1}{\omega_{3}}-\frac{\varpi_{1}^{FD}}{\omega_{3}\varpi_{1}^{HD}}\right)\right)\\ & +\frac{\varpi_{1}^{FD}}{\omega_{3}+\varpi_{1}^{FD}}\exp\left(\frac{-1}{\varpi_{1}^{FD}}-\left(\frac{\varpi_{1}^{FD}}{\varpi_{1}^{HD}}-1\right)\left(\frac{1}{\varpi_{1}^{FD}}+\frac{1}{\omega_{3}}\right)\right), \end{array}$$

where $\varpi_{1}^{HD}=\min\left(\frac{\omega_{4}}{\gamma_{0}^{u2,HD}}-\omega_{1},\frac{\omega_{1}}{\gamma_{0}^{u1,HD}}\right)-\omega_{2}$.

Since the channel gains follow exponent distribution, the result is derived. It can be noted that since $\gamma_{0}^{u1,HD}\geq \gamma_{0}^{u1}$ and $\gamma_{0}^{u2,HD}\geq \gamma_{0}^{u2}$, it implies $\frac{\varpi_{1}^{FD}}{\varpi_{1}^{HD}}\geq 1$. Hence, we have $\varpi_{1}^{HD}\geq \varpi_{1}^{FD}$, that leads to the non-negative value of $\frac{\varpi_{1}^{FD}}{\varpi_{1}^{HD}}-1$. And $\Psi_{u1-2}^{A-I}=\mathrm{Pr}\left(\gamma_{\mathrm{bs}\to \mathrm{re}}^{u2}\geq \gamma_{0}^{u2}\right)=OP_{u2-1}^{FD}$. The proposition is thus, obtained. This is end of proof.

## Appendix F. Proof of Proposition 5

The first part in (33) can be obtained as

(A24) $$\begin{array}{cc} OP_{u2-1}^{A-I}= & \mathrm{Pr}\left(\phi \right)\mathrm{Pr}\left(\gamma_{\mathrm{re}\to u2}^{u2}<\gamma_{0}^{u2}\right)\\ = & \Psi_{\phi }\times \left(1-OP_{u2-2}^{FD}\right). \end{array}$$

The second term in (33) is determined as

(A25) $$\begin{array}{cc} OP_{u2-2}^{A-I}= & \mathrm{Pr}\left(\left(1-\gamma_{\mathrm{bs}\to \mathrm{re}}^{u2,HD}\geq \gamma_{0}^{u2,HD}\cap \gamma_{\mathrm{re}\to u2}^{u2,HD}\geq \gamma_{0}^{u1,HD}\right)\cap \left(1-\phi \right)\right)\\ = & \underset{=OP_{u2}^{HD}}{\underset{︸}{1-\mathrm{Pr}\left(\gamma_{\mathrm{bs}\to \mathrm{re}}^{u2,HD}\geq \gamma_{0}^{u2,HD}\cap \gamma_{\mathrm{re}\to u2}^{u2,HD}\geq \gamma_{0}^{u1,HD}\right)}}\\ & -\mathrm{Pr}\left(\phi \right)+\mathrm{Pr}\left(\gamma_{\mathrm{bs}\to \mathrm{re}}^{u2,HD}\geq \gamma_{0}^{u2,HD}\cap \gamma_{\mathrm{re}\to u2}^{u2,HD}\geq \gamma_{0}^{u1,HD}\cap \phi \right)\\ = & OP_{u2}^{HD}-\Psi_{\phi }+\Psi_{u2}^{A-I}. \end{array}$$

The last term in (A25) can be determined as

(A26) $$\begin{array}{cc} \Psi_{u2}^{A-I}\overset{\Delta }{=} & \mathrm{Pr}\left(\gamma_{\mathrm{re}\to u2}^{u2,HD}\geq \gamma_{0}^{u1,HD}\right)\mathrm{Pr}\left(\gamma_{\mathrm{bs}\to u1}^{u2}\geq \gamma_{0}^{u2}\cap \gamma_{\mathrm{bs}\to u1}^{u1}\geq \gamma_{0}^{u1}\right)\\ & \mathrm{Pr}\left(\gamma_{\mathrm{bs}\to \mathrm{re}}^{u2,HD}\geq \gamma_{0}^{u2,HD}\cap \gamma_{\mathrm{bs}\to \mathrm{re}}^{u2}\geq \gamma_{0}^{u2}\right)\\ = & \Psi_{u2-1}^{A-I}\times \left(1-OP_{u1}^{FD}\right)\times \Psi_{u2-2}^{A-I}. \end{array}$$

with

(A27) $$\begin{array}{ccc} \Psi_{u2-1}^{A-I}\overset{\Delta }{=} & \mathrm{Pr}\left(\gamma_{\mathrm{re}\to u2}^{u2,HD}\geq \gamma_{0}^{u1,HD}\right)= & \left\{\begin{array}{cc} 0, & \upsilon_{2}^{HD}\leq 0,\\ \exp\left(-\frac{-1}{\upsilon_{2}^{HD}}\right), & \upsilon_{2}^{HD}>0. \end{array}\right. \end{array}$$

and

(A28) $$\begin{array}{cc} \Psi_{u2-2}^{A-I}= & \mathrm{Pr}\left({\left|h_{\mathrm{bs},\mathrm{re}}\right|}^{2}\geq \frac{\lambda_{\mathrm{bs},\mathrm{re}}}{\upsilon_{1}^{HD}}\cap {\left|h_{\mathrm{bs},\mathrm{re}}\right|}^{2}\geq \frac{\varphi_{3}\lambda_{\mathrm{bs},\mathrm{re}}}{\upsilon_{1}^{FD}\lambda_{\mathrm{re},\mathrm{re}}}{\left|{\hat{f}}_{\mathrm{re},\mathrm{re}}\right|}^{2}+\frac{\lambda_{\mathrm{bs},\mathrm{re}}}{\upsilon_{1}^{FD}}\right)\\ = & \exp\left(\frac{-1}{\upsilon_{1}^{HD}}\right)\left(1-\exp\left(\frac{1}{\varphi_{3}}-\frac{\upsilon_{1}^{FD}}{\varphi_{3}\upsilon_{1}^{HD}}\right)\right)+\frac{\upsilon_{1}^{FD}}{\varphi_{3}+\upsilon_{1}^{FD}}\exp\left(\frac{\upsilon_{1}^{FD}}{\varphi_{3}\upsilon_{1}^{HD}}+\frac{1}{\varphi_{3}}-\frac{1}{\upsilon_{1}^{HD}}\right), \end{array}$$

if $\min\left(\upsilon_{1}^{FD},\upsilon_{1}^{HD}\right)>0$ and $\Psi_{u2-2}^{A-I}=0$ if $\min\left(\upsilon_{1},\vartheta_{2}\right)\leq 0$ with $\upsilon_{1}^{HD}\overset{\Delta }{=}\frac{\varphi_{1}}{\gamma_{0}^{u2,HD}}-\varphi_{2}$, $\upsilon_{1}^{FD}\overset{\Delta }{=}\frac{\varphi_{1}}{\gamma_{0}^{u2}}-\varphi_{2}$.
